# Lower creatinine levels are associated with an increased risk of depression: evidence from the China Health and Retirement Longitudinal Study

**DOI:** 10.3389/fpsyt.2025.1446897

**Published:** 2025-02-25

**Authors:** Fajin Liu, Xiaogang Zhong, Cuiting Wang

**Affiliations:** ^1^ Department of Neurological Rehabilitation, The Affiliated Rehabilitation Hospital of Chongqing Medical University, Chongqing, China; ^2^ College of Rehabilitation Medicine, Chongqing Medical University, Chongqing, China; ^3^ Department of Neurological Rehabilitation, Rehabilitation Hospital of, Chongqing, China; ^4^ NHC Key Laboratory of Diagnosis and Treatment on Brain Functional Diseases, The First Affiliated Hospital of Chongqing Medical University, Chongqing, China

**Keywords:** depression, creatinine, association, Chinese, CHARLS, MENDA

## Abstract

**Introduction:**

Previous studies have found that depressive patients tend to have low levels of creatinine; however, the extent to which creatinine levels are associated with depression has been poorly investigated. Therefore, this study aimed to explore the relationship between creatinine levels and depression.

**Methods:**

The participants and follow-up data from the China Health and Retirement Longitudinal Study (CHARLS), as well as metabolomics data from the Metabolite Network of Depression Database (MENDA), were collected. The 10-item Center for Epidemiologic Studies Depression Scale (CESD-10) was used to assess the severity of depression. Spearman correlation analysis, spline regression, and binary logistic regression models were employed to explore the relationship between creatinine levels and depression.

**Results:**

A total of 7,826 participants and 3,886 follow-up participants were included in the CHARLS 2011 and 2015 surveys. Of these, 37.9% (2,966/7,826) and 34.6% (13,44/3,886) of participants experienced depression in CHARLS 2011 and 2015, respectively. The creatinine level was negatively correlated with the total CESD-10 score and dimensions scores, showing an inverse dose–response relationship between creatinine levels and depression. Compared with participants with high creatinine levels, those with middle creatinine levels were associated with a higher risk of depression (OR = 1.22, 95% CI = 1.08–1.38), while participants with low creatinine levels had the highest risk of depression (OR = 1.30, 95% CI = 1.13–1.49) in the fully adjusted model. Similar results were observed in the follow-up data, and the MENDA metabolomics data validated the negative correlation between creatinine levels and the severity of depression.

**Conclusion:**

Lower levels of creatinine were closely associated with a higher risk of depression, and it could serve as a potential marker for identifying individuals at high risk of depression.

## 1 Introduction

Depression is a serious mental illness characterized by persistent sadness and loss of interest (1), which significantly impacts the quality of life. Previous studies have shown that the lifetime prevalence of depression is 6.8%–20.6% (2, 3), and it has become one of the leading causes of years lived with disability worldwide (4). As depression and depressive symptoms are dynamic and changeable throughout a person’s life, they are common in the population, especially among middle-aged and elderly adults (5). As China gradually transitions into an aging society, this challenge will become increasingly serious.

In recent years, some available and economical blood biomarkers have been proposed (6–8). Creatinine, a metabolite in the blood, comes from normal muscle wear and tear and can be filtered through the glomerulus and then excreted in urine. It is widely used in the evaluation of renal function, such as the glomerular filtration rate. Some studies have found that depression is related to a decline in renal function (9–11). In addition, creatinine levels are parallel to muscle mass, so they can also be used as an evaluation indicator of muscle mass (12, 13). Some studies have also found that muscle loss may be a risk factor for depression (14, 15). Nevertheless, in most of these studies, creatinine has been used as an indicator to evaluate renal function and sarcopenia, and the direct relationship between creatinine and depression has not been clarified.

Furthermore, increasing evidence shows that energy metabolism is disturbed in depressive patients (15–17). Creatine, an important intermediate of energy metabolism, is widely involved in the recycling process of adenosine phosphate (ATP) (18–20). Importantly, creatinine is the metabolite of creatine, so we speculate that creatinine may be closely related to depression. Some metabolomic evidence has emerged (21–23). For example, different teams from China both found that creatinine levels were downregulated in the urine of chronic unpredictable mild stress rats (21, 23). Interestingly, urine creatinine levels were increased in depression patients after being treated with antidepressants (22). More importantly, creatinine levels are a common indicator that can be directly accessed from the blood in clinical practice. Thus, this study aims to explore the relationship between creatinine and depression through the China Health and Retirement Longitudinal Study (CHARLS).

## 2 Methods

### 2.1 Study population and design

CHARLS is an ongoing national longitudinal survey designed to better understand the socioeconomic determinants and consequences of aging. The survey collects comprehensive data on demographics, physical and psychosocial health, and socioeconomic factors (24). In the CHARLS baseline survey, participants were selected using multi-stage probability sampling. Specifically, 150 counties were randomly selected from 28 provinces, and three villages or communities in each county were selected using the probability-proportional-to-size (PPS) sampling technique (24). The initial sample of CHARLS (wave 1: 2011–2012) includes 17,708 individuals, with follow-up occurring every 2 years (25). Furthermore, the study adhered to the Strengthening the Reporting of Observational Studies in Epidemiology (STROBE) reporting guideline. More detailed information is available on the CHARLS project website (http://charls.pku.edu.cn/). We downloaded the harmonized data and blood data from the official website for further analysis.

### 2.2 Demographic covariates

To minimize bias, demographic covariates were also included for adjustment when evaluating the association between creatinine and depression. These covariates included age, gender, body mass index (BMI), education level, marital status, residence, smoking, drinking, hypertension, diabetes, heart problems, kidney problems, and liver problems, all of which were extracted from the questionnaire. Education level was categorized as “less than lower secondary”, “upper secondary and vocal training”, and “tertiary”. Marital status was categorized as “married” and “unmarried”. Residence was categorized as “urban” and “rural”. Health-related behaviors included whether the respondent reported ever smoking (“no”, “yes”) or drinking any alcohol before (“no”, “yes”). Disease histories included whether the respondent reported being diagnosed with hypertension, diabetes, heart, kidney, or liver problems (“no”, “yes”).

### 2.3 Serum creatinine

**Figure 1:**
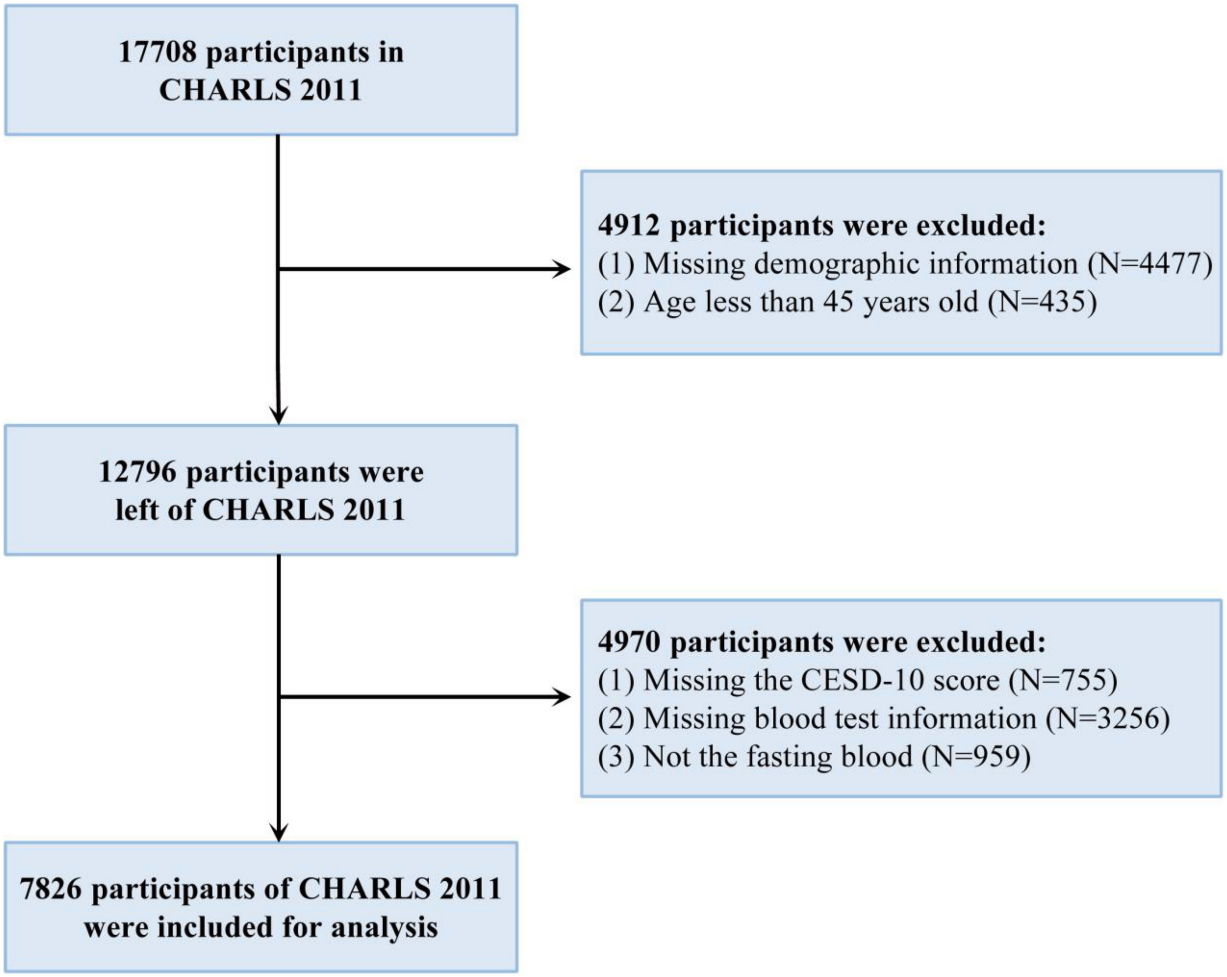
The flowchart of the included participants in wave 1. CHARLS, China Health and Retirement Longitudinal Study; CESD-10, 10-item center for epidemiologic studies depression scale.

The well-trained staff of the China Center for Disease Control and Prevention (CDC) collected venous blood from each participant and sent it to the local laboratory at 4°C. The blood samples were separated into plasma and buffy coat, frozen at − 20°C, and sent to China CDC in Beijing within 2 weeks. Finally, they were stored at − 80°C until tested at the Youanmen Center for Clinical Laboratory of Capital Medical University. Serum creatinine was measured using a rate-blanked and compensated Jaffe creatinine method (26). Notably, CHARLS only collected blood samples in 2011 (wave 1) and 2015 (wave 3), so this study includes only the 2011 baseline data and 2015 follow-up data. The participants from wave 1 are shown in **Figure 1**.

### 2.4 Definition of depression

Depression was evaluated using the 10-item Center for Epidemiological Studies Depression Scale (CESD-10). The scale consists of 10 items, each rated on a 3-point (0, 1, 2, 3) Likert scale. Responses varied from “rarely or none of the time (< 1 day)”, “some or a little of the time (1–2 days)”, “occasionally or a moderate amount of time (3–4 days)”, and “most or all of the time (5–7 days)”, respectively. Therefore, the total score on the CESD-10 scale ranged from 0 to 30. Depression was considered present when the cumulative score was ≥ 10 (11, 27, 28). The CESD-10 scale has been shown to have good reliability and validity in the Chinese population (29).

### 2.5 Inclusion and exclusion criteria

For this study, we used data from CHARLS in 2011 and 2015. The inclusion criteria were as follows: (1) age ≥ 45 years old; (2) availability of completed blood test information; and (3) availability of completed CESD-10 information. The exclusion criteria were as follows: (1) age < 45 years; (2) lack of basic demographic information; (3) lack of blood test information; (4) nonfasting blood test; and (5) missing CESD-10 information.

### 2.6 Statistical analysis

All statistical analyses were performed in R (version 4.1.0) and SPSS (version 25.0). Mean and standard deviation were used to describe quantitative data, while frequency and percentage were used to describe qualitative data. Spearman correlation coefficient was used to explore the correlation between creatinine levels and CESD-10 scores. The dose–response relationship between creatine levels and depression was preliminarily examined using spline regression models. Subsequently, creatinine levels were divided into three groups (< 0.689, 0.689–0.836, and ≥ 0.836 mg/dL) according to the tertiles. The *t*-test, one-way ANOVA, or Chi-square test was used to describe the difference between the groups.

A binary logistic regression model was used to explore the relationship between creatinine levels and depression. Model 1 was an unadjusted model; model 2 adjusted for the basic demographic information, including age, gender, BMI, education level, marital status, and residence; model 3 adjusted for health-related behaviors, including drinking and smoking; model 4 adjusted for disease history, including hypertension, diabetes, heart problems, kidney problems, and liver problems; model 5 was the fully adjusted model, including age, gender, education level, marital status, residence, drinking, smoking, hypertension, diabetes, heart problems, kidney problems, and liver problems. A *p-*value < 0.05 (two-sided) was considered to be statistically significant.

## 3 Results

### 3.1 Basic information

As described in the **Figure 1**, a total of 7,826 participants were included in the main analysis. The mean age of the participants was 59.11 years ± 9.23 years old; 3,681 (47.0%) participants were men. Most of the participants had an education level of less than lower secondary, were married, lived in rural areas, and did not have a history of smoking, drinking, hypertension, diabetes, heart problems, kidney problems, or liver problems (**Table 1**). A total of 37.9% (2,966/7,826) of the participants experienced depression.

**Table 1:** Demographic characteristics of the included participants.

Variables	Total (*n* = 7,826)	Q1 (*n* = 2,492)	Q2 (*n* =2,784)	Q3 (*n* = 2,550)	*p-*value
Age	59.11 ± 9.23	57.42 ± 8.69	58.94 ± 9.08	60.95 ± 9.57	< 0.001
BMI	24.11 ± 29.63	23.85 ± 9.04	24.71 ± 47.78	23.72 ± 11.01	0.412
Sex					
Men	3,681 (47.0)	408 (16.4)	1,230 (44.2)	2,043 (80.1)	< 0.001
Women	4,145 (53.0)	2,084 (83.6)	1,554 (55.8)	507 (19.9)	
Education level					
Low	7,054 (90.1)	2,311 (92.7)	2,501 (89.8)	2,242 (87.9)	< 0.001
Middle	683 (8.7)	166 (6.7)	252 (9.1)	265 (10.4)	
High	89 (1.1)	15 (0.6)	31 (1.1)	43 (1.7)	
Marital status					
Married	6,563 (83.9)	2,054 (82.4)	2,318 (83.3)	2,191 (85.9)	0.002
Unmarried	1,263 (16.1)	438 (17.6)	466 (16.7)	359 (14.1)	
Residence					
Urban	2,848 (36.4)	824 (33.1)	998 (35.8)	1,026 (40.2)	< 0.001
Rural	4,978 (63.6)	1,668 (66.9)	1,786 (64.2)	1,524 (59.8)	
Smoking					
No	4,728 (60.4)	2,032 (81.5)	1,724 (61.9)	972 (38.1)	< 0.001
Yes	3,098 (39.6)	460 (18.5)	1,060 (38.1)	1,578 (61.9)	
Drinking					
No	4,744 (60.6)	1,885 (75.6)	1,720 (61.8)	1,139 (44.7)	< 0.001
Yes	3,082 (39.4)	607 (24.4)	1,064 (38.2)	1,411 (55.3)	
Hypertension					
No	5,718 (73.1)	1,902 (76.3)	2,067 (74.2)	1,749 (68.6)	< 0.001
Yes	2,108 (26.9)	590 (23.7)	717 (25.8)	801 (31.4)	
Diabetes					
No	7,326 (93.6)	2,334 (93.7)	2,611 (93.8)	2,371 (93.4)	0.821
Yes	500 (6.4)	158 (6.3)	173 (6.2)	169 (6.6)	
Heart problems					
No	6,876 (87.9)	2,228 (89.4)	2,435 (87.5)	2,213 (86.8)	0.013
Yes	950 (12.1)	264 (10.6)	349 (12.5)	337 (13.2)	
Kidney problems					
No	7,361 (94.1)	2,373 (95.2)	2,628 (94.4)	2,360 (92.5)	< 0.001
Yes	465 (5.9)	119 (4.8)	156 (5.6)	190 (7.5)	
Liver problems					
No	7,556 (96.5)	2,413 (96.8)	2,692 (96.7)	2,451 (96.1)	0.334
Yes	270 (3.5)	79 (3.2)	92 (3.3)	99 (3.9)	
CESD-10 scale					
< 10	4,860 (62.1)	1,413 (56.7)	1,696 (60.9)	1,751 (68.7)	< 0.001
≥10	2,966 (37.9)	1,079 (43.3)	1,088 (39.1)	799 (31.3)	

### 3.2 Correlation analysis and dose–response analysis

**Figure 2:**
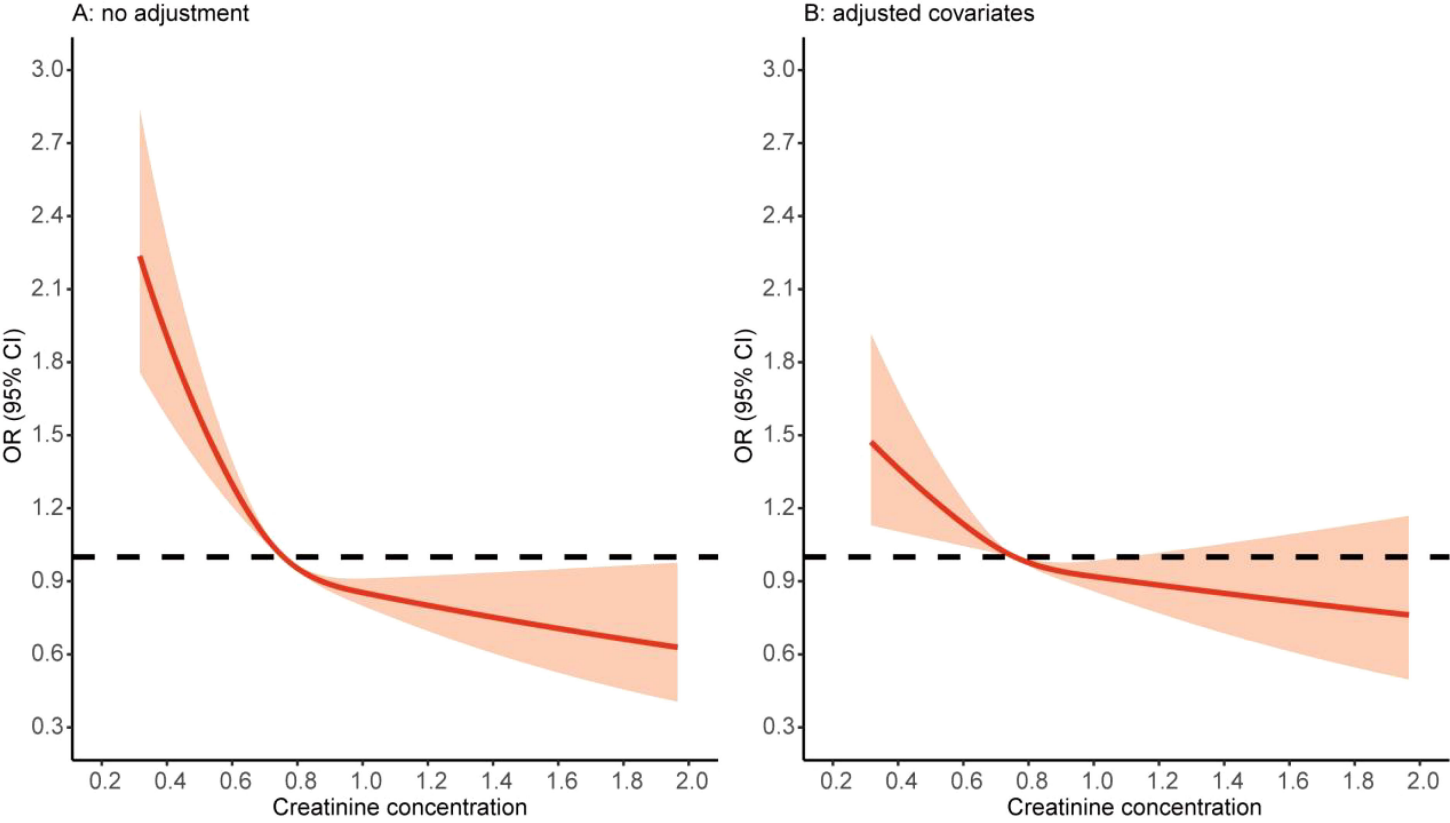
Restricted cubic spline of the creatinine and depression. **(A)** Without any adjustment of demographic covariates. **(B)** Adjustment for all demographic covariates, including age, sex, BMI, education level, marital status, residence, smoking, drinking, hypertension, diabetes, heart problems, kidney problems, and liver problems. OR, odds ratio; CI, confidence interval.

Correlation analysis results showed that the creatinine level was negatively correlated with the total score and each dimension score of the CESD-10 scale (*p* < 0.05, **Supplementary Figure S1**). We then used a spline regression model to preliminary explore the shape of the association between creatinine levels and depression. The restricted cubic spline function used the default three knots (at the 10th, 50th, and 90th percentiles). Both the unadjusted and fully adjusted models showed a dose–response relationship between the creatinine levels and depression (**Figure 2**). Therefore, we divided the participants into groups according to the tertiles of creatinine levels for further analysis.

### 3.3 Univariate analysis

Univariate analysis showed a significant statistical significance in age, gender, education level, marital status, residence, drinking, smoking, hypertension, heart problems, and kidney problems. Compared with the lower creatinine level group, the higher creatinine level group tended to be older, have a higher proportion of men, have higher education, be married, live in urban areas, and have a history of smoking, drinking, hypertension, heart problems, and kidney problems (**Table 1**). There was no statistical significance for BMI, diabetes, and liver problems (*p* > 0.05).

**Figure 3:**
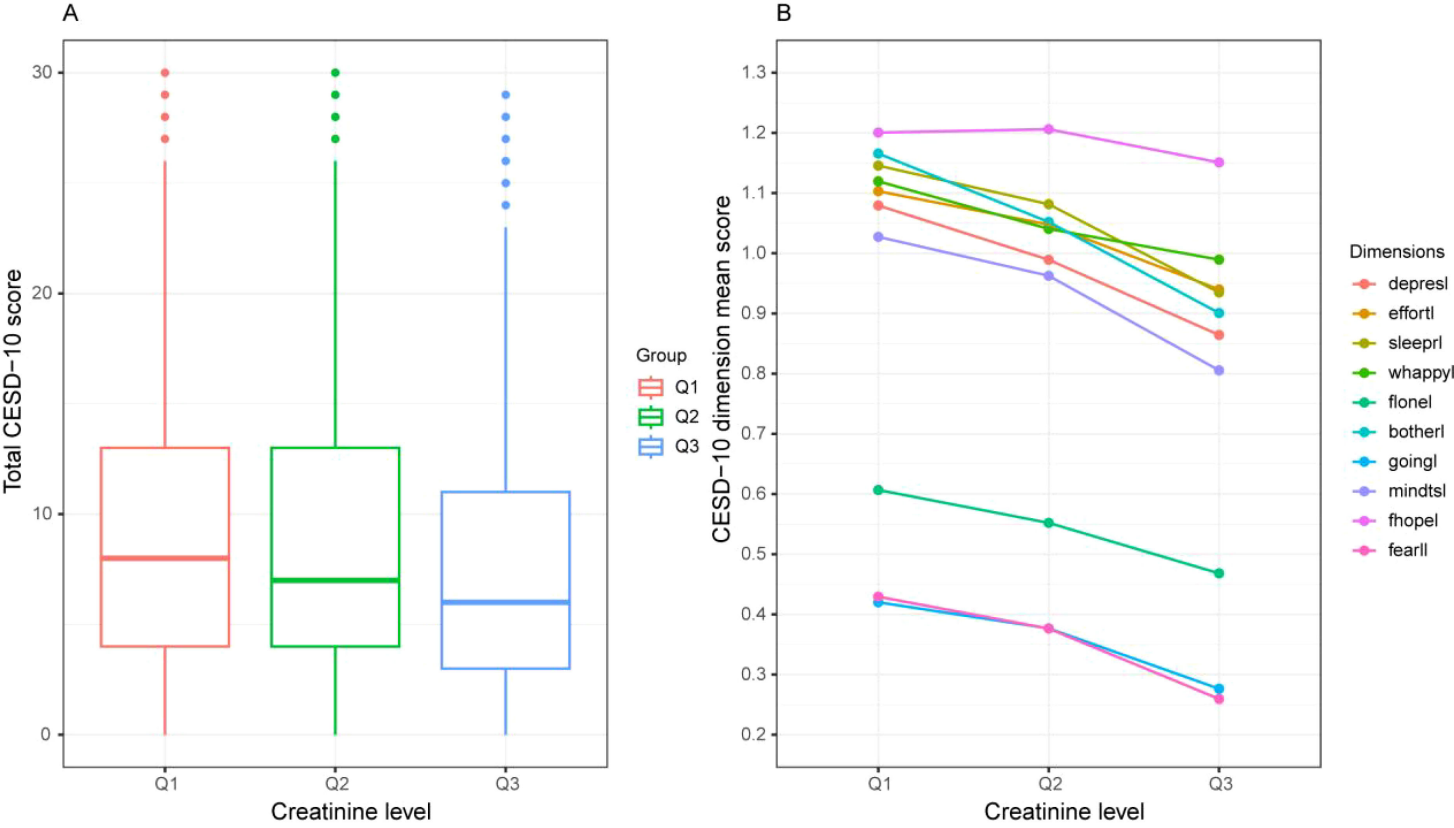
CESD-10 score tendency among three groups according to the tertiles of creatinine level. **(A)** Total CESD-10 score and creatinine level. **(B)** Dimension score of CESD-10 and creatinine. CESD-10, 10-item center for epidemiological studies depression scale.

Compared with the lower creatinine level group, the one-way ANOVA results showed that the higher creatinine level group had a lower depression score (**Table 2**), regardless of the total CESD-10 score or each dimension score (**Figure 3**). Almost all of the dimension items showed a downward trend (*p* < 0.05). Moreover, the proportion of participants with depression was significantly lower (**Table 1**).

**Table 2:** The CESD-10 score comparison between the groups.

Items	Total (*n* = 7,826)	Q1 (*n* = 2,492)	Q2 (*n* = 2,784)	Q3 (*n* = 2,550)	*p-*value
Total score	8.52 ± 6.35	9.30 ± 6.57	8.69 ± 6.43	7.59 ± 5.91	< 0.001
Depresl	0.98 ± 1.07	1.08 ± 1.10	0.99 ± 1.06	0.86 ± 1.04	< 0.001
Effortl	1.03 ± 1.15	1.10 ± 1.15	1.05 ± 1.15	0.94 ± 1.14	< 0.001
Sleeprl	1.05 ± 1.19	1.15 ± 1.21	1.08 ± 1.20	0.93 ± 1.17	< 0.001
Whappyl	1.05 ± 1.11	1.12 ± 1.13	1.04 ± 1.10	0.99 ± 1.11	< 0.001
Flonel	0.54 ± 0.95	0.61 ± 1.00	0.55 ± 0.96	0.47 ± 0.90	< 0.001
Botherl	1.04 ± 1.11	1.17 ± 1.13	1.05 ± 1.11	0.90 ± 1.08	< 0.001
Goingl	0.36 ± 0.79	0.42 ± 0.84	0.38 ± 0.80	0.28 ± 0.70	< 0.001
Mindtsl	0.93 ± 1.09	1.03 ± 1.11	0.96 ± 1.10	0.81 ± 1.04	< 0.001
Fhopel	1.19 ± 1.21	1.20 ± 1.22	1.21 ± 1.20	1.15 ± 1.21	0.194
Fearl	0.36 ± 0.78	0.43 ± 0.86	0.38 ± 0.80	0.26 ± 0.67	< 0.001

### 3.4 Source of depression

In addition, we also explored the source dimensions of depression. The mean value of the total score on the CESD-10 scale was 0.852, while the items Fearl-10, Goingl-7, and Flonel-5 were far lower than the threshold. In contrast, the items Fhopel-9, Sleeprl-3, and Whappyl-4 were far higher than the threshold, indicating that the depression in middle-aged and elderly adults in China mainly stemmed from the items Fhopel-9, Sleeprl-3, and Whappyl-4.

### 3.5 Regression results

The logistic regression model results are shown in **Table 3**. In the unadjusted model, participants with lower creatinine levels were more likely to suffer from depression (middle level: OR = 1.41, 95% CI = 1.26–1.57; low level: OR = 1.67, 95% CI = 1.49–1.88). To exclude the influence of confounding covariates, models 2, 3, and 4 adjusted for demographic information, health-related behaviors, and disease histories, respectively, and the results were consistent with those of the crude model. In model 5 (fully adjusted model), compared with the higher creatinine level group, the OR of the middle-level group was 1.22 (95% CI = 1.08-1.38), and for the low-level group, it was 1.30 (95% CI = 1.13–1.49), respectively. The *p*-values for the trend in all the models were statistically significant. The above results indicate that a lower creatinine level is significantly associated with a higher risk of depression.

**Table 3:** Logistic regression model on creatinine level and depression.

Items	*n*	Events (%)	OR (95% CI)				
			Model 1	Model 2	Model 3	Model 4	Model 5
Creatinine							
High	2,550	799 (31.3)	Reference	Reference	Reference	Reference	Reference
Middle	2,784	1,088 (39.1)	1.41 (1.26–1.57)	1.17 (1.03–1.32)	1.33 (1.18–1.49)	1.46 (1.30–1.64)	1.22 (1.08–1.38)
Low	2,492	1,079 (43.3)	1.67 (1.49–1.88)	1.21 (1.05–1.39)	1.51 (1.34–1.71)	1.78 (1.59–2.00)	1.30 (1.13–1.49)
*p*-value for trend			< 0.001	< 0.001	< 0.001	< 0.001	0.001

Model 1: unadjusted model. Model 2: adjusted for demographic information. Model 3: adjusted for health-related behaviors. Model 4: adjusted for disease histories. Model 5: fully adjusted model.

### 3.6 Follow-up results

In the follow-up data of CHARLS 2015 (wave 3), a total of 3,886 participants with blood tests were included (**Supplementary Table S1**). Of these, 34.6% (1,344/3,886) of the participants experienced depression. Consistent with the above results, the total CESD-10 score decreased as the creatinine level increased (**Supplementary Table S2**). Depression was primarily associated with the dimension items Fhopel-5, Sleeprl-7, and Whappyl-8 (**Supplementary Figure S2**). Both the crude model and adjusted model found that lower creatinine level was significantly associated with a higher risk of depression (**Supplementary Table S3**).

### 3.7 Comparison of wave 1 and wave 3

Based on the depression measured in wave 1 and wave 3, we divided the participants into four groups: nondepression (NN), depression-relief (YN), new-onset depression (NY), and persistent depression (YY), and compared their CESD-10 score and creatinine levels (**Supplementary Figure S3**). The paired *t*-test showed that the creatinine levels of participants in all four groups in 2015 were generally significantly higher than those in 2011 (*p* < 0.05), which was consistent with the fact that the depression rate in 2015 was lower than that in 2011. The CESD-10 score in the YN and NY groups in 2015 was significantly different from 2011 (*p* < 0.01), while there was no statistical difference between the NN and YY groups (*p* > 0.05). Furthermore, we compared the CESD-10 score and creatinine level of each group in 2015. The results showed that the CESD-10 score of the YN group was significantly higher than that of the NN group (*p* < 0.01), and the creatinine level was significantly lower than that of the NN group (*p* = 0.03), indicating that the YN group had more severe depression, and the creatinine level had not returned to normal. Meanwhile, the CESD-10 score of the YY group was significantly higher than that of the NY group (*p* < 0.01), and the creatinine level was significantly lower in the YY group (*p* = 0.03), indicating the participants with a long-term depression course exhibited lower creatinine levels and more severe depression.

Furthermore, we explored the correlation of creatinine concentration between wave 1 and wave 3. The Spearman analysis results showed a correlation coefficient of 0.70 (*p* < 0.001, **Supplementary Figure S4**), which indicates that the baseline and follow-up creatinine levels were consistent and stable.

### 3.8 Clinical metabolomics validation

Furthermore, we validated the correlation between creatinine levels and the severity of depression at the metabolomics level by downloading and analyzing creatinine metabolic data and Hamilton Depression Scale (HAMD) scores of major depressive disorder (MDD) patients from the Metabolite Network of Depression Database (MENDA, http://menda.cqmu.edu.cn:8080/index.php) (30). Correlation analysis showed that the creatinine level was significantly negatively correlated with the HAMD score both in the plasma (*r* = − 0.40, *p* < 0.001) and urine (*r* = − 0.44, *p* < 0.001) of adult MDD patients. Additionally, we observed that the creatinine level was significantly negatively correlated with the HAMD score in the plasma (*r* = − 0.84, *p* < 0.001) of MDD patients in children and adolescents (**Supplementary Figure S5**).

## 4 Discussion

Depression is a mental disease that seriously damages the social function of patients (31, 32) and is widespread among middle-aged and elderly adults (25, 28). However, depression is often overlooked and undertreated, as patients are frequently burdened with other chronic conditions, such as hypertension, diabetes, and heart disease (33–35). In addition, some studies have found that the effect of antidepressants on young people is significantly better than on the elderly (36, 37). As China becomes the world’s fastest aging country, there is an urgent need to closely address depression in middle-aged and elderly adults.

In this study, we found that serum creatinine concentration was negatively correlated with depression, and lower creatinine levels were associated with a higher risk of depression, based on data from a large, nationally representative cohort of middle-aged and elderly adults in China. Even after adjusting for many possible confounding variables, our study still provides convincing results. To our knowledge, this is the first study to explore the relationship between creatinine and depression in this population.

Creatine is a component of vertebrate muscle, synthesized in the liver and transported throughout the body, where it is then phosphorylated to store energy for muscle contraction. It is widely involved in the recycling process of adenosine phosphate (18–20) and is closely related to depression (19). For example, animal models of depression have found that creatine is downregulated in various tissues, such as the hippocampus, prefrontal lobe, plasma, serum, and urine (38–42). In addition, Zhou et al. observed that creatine was downregulated in the plasma of children and adolescents with depression through metabolomics (43). While exogenous supplementation of creatine or ATP can alleviate depression (44, 45), the possible explanation is that creatine can accelerate the clinical response to selective serotonin reuptake inhibitors, promote the differentiation of neurons, and thus improve the overall efficacy (17, 18, 46). Therefore, creatine’s effect on energy metabolism may be part of the basis for its treatment of depression.

Creatinine is the crystal end product of creatine metabolism, which exists in urine, muscle, and blood. Serum creatinine is the product of human muscle metabolism. Previous studies have found that creatinine is disturbed in depression. For example, Peng et al. found that the creatinine level in young depressive patients was decreased (47). Paradoxically, Lee et al. found that the creatinine level in Korean female depressive patients was increased, with no significant change in men (48). This could be due to the differences in eating habits and geography. Nevertheless, the vast majority of studies favored a lower creatinine level in depressive patients. For example, Pu et al. found a significant decrease in creatinine in the peripheral blood of depressive patients through a meta-analysis (7). Zhou et al. found a significant decrease in creatinine in the plasma of children and adolescents with depression through metabolomics (43). Using a data-driven approach to integrate metabolomics data from existing animal models of depression, Pu et al. found that creatinine was decreased in various tissues (19). After the antidepressant intervention, the creatinine or creatine levels in both blood and urine were significantly upregulated (49–51). Moreover, Niklasson et al. found that the creatinine level in cerebrospinal fluid was negatively correlated with suicidal ideation in depressive patients (52). Consistent with the above findings, we found that creatinine level was negatively correlated with CESD-10 score and HAMD score and that there was a dose–response relationship between creatinine level and depression. This association persisted even after we adjusted for demographic information, health-related behaviors, and disease histories. These results suggest that the creatinine level is disturbed in depression, regardless of tissue type, age, and species, and that creatinine may play a key role in the development of depression.

Moreover, we also explored the sources of depression and found that positive factors and sleep restlessness were the main sources of depression. According to previous studies, poor sleep is common among middle-aged and elderly adults (53, 54), and it has exceeded 20% in China (55, 56). It is well-known that sleep deprivation can lead to decreased alertness and difficulty in performing mental tasks (18), whereas subjects treated with creatine showed a lower decay of psychomotor activity attenuation and improved mood in tests (57, 58). These studies illustrate the effectiveness of creatine in improving sleep deprivation and mental fatigue, providing some evidence for guiding the alleviation of depression among middle-aged and elderly adults in China.

## 5 Conclusion

Taken together, our study provides evidence that a lower level of creatinine is independently associated with a higher risk of depression in Chinese middle-aged and elderly adults. These findings may contribute to the further development of depression biomarkers.

## 6 Limitations

There are also some limitations in this study. Firstly, we could not determine the causal relationship between creatinine and depression. Secondly, depression was assessed using the CESD-10, a subjective self-assessment scale, instead of evaluation by a professional psychiatry doctor. Thirdly, although we adjusted for some confounding variables, there may be other factors, such as the family histories of depression and renal function, leading to potential residual bias. Fourthly, body composition and physical exercise may affect creatinine levels; however, the cohort did not provide this information. Therefore, the impact of these factors on depression warrants further research. Fifthly, muscles not only produce creatinine but also brain-derived neurotrophic factor (BDNF), which is significantly associated with depression. Thus, BDNF should also be considered in subsequent studies. Sixthly, a longer follow-up period is needed to confirm the relationship between creatinine and depression. Lastly, further studies are required to explore the molecular mechanisms linking creatinine and depression.

## Data Availability

The data in this study were publicly available datasets, which can be found at http://charls.pku.edu.cn/.
