# Supplementary material for: Lower creatinine levels are associated with an increased risk of depression: evidence from the China Health and Retirement Longitudinal Study
Source: Front Psychiatry. 2025 Feb 25;16:1446897. doi: 10.3389/fpsyt.2025.1446897 (PMC11894454; doi:10.3389/fpsyt.2025.1446897)
Supplement: Supplementary file 1 [file SupplementaryFile1.docx]

**SUPPLEMENTARY TABLE 1** Demographic characteristics of the follow-up participants.

| **Items** | **Total (n=3886)** | **Q1 (n=1302)** | **Q2 (n=1275)** | **Q3 (n=1309)** | ***P* value** |
| --- | --- | --- | --- | --- | --- |
| Age | 62.09±8.30 | 60.34±7.76 | 61.85±7.99 | 64.08±8.69 | <0.001 |
| BMI | 24.55±17.20 | 25.29±20.32 | 24.66±21.59 | 23.70±3.60 | 0.058 |
| Sex |  |  |  |  | <0.001 |
| Male | 1815 (46.7) | 152 (11.7) | 607 (47.6) | 1056 (80.7) |  |
| Female | 2071 (53.3) | 1150 (88.3) | 668 (52.4) | 253 (19.3) |  |
| Education level |  |  |  |  | <0.001 |
| Low | 3526 (90.7) | 1225 (94.1) | 1143 (89.6) | 1158 (88.5) |  |
| Middle | 324 (8.3) | 73 (5.6) | 111 (8.7) | 140 (10.7) |  |
| High | 36 (0.9) | 4 (0.3) | 21 (1.6) | 11 (0.8) |  |
| Marital status |  |  |  |  | 0.882 |
| Married | 3224 (83.0) | 1075 (82.6) | 1062 (83.3) | 1087 (83.0) |  |
| Unmarried | 662 (17.0) | 227 (17.4) | 213 (16.7) | 222 (17.0) |  |
| Residence |  |  |  |  | 0.194 |
| Urban | 1310 (33.7) | 444 (34.1) | 406 (31.8) | 460 (35.1) |  |
| Rural | 2576 (66.3) | 858 (65.9) | 869 (68.2) | 849 (64.9) |  |
| Smoking |  |  |  |  | <0.001 |
| No | 2187 (56.3) | 1080 (82.9) | 692 (54.3) | 415 (31.7) |  |
| Yes | 1699 (43.7) | 222 (17.1) | 583 (45.7) | 894 (68.3) |  |
| Drinking |  |  |  |  | <0.001 |
| No | 2101 (54.1) | 947 (72.7) | 670 (52.5) | 484 (37.0) |  |
| Yes | 1785 (45.9) | 355 (27.3) | 605 (47.5) | 825 (63.0) |  |
| Hypertension |  |  |  |  | 0.005 |
| No | 2452 (63.1) | 854 (65.6) | 817 (64.1) | 781 (59.7) |  |
| Yes | 1434 (36.9) | 448 (34.4) | 458 (35.9) | 528 (40.3) |  |
| Diabetes |  |  |  |  | <0.001 |
| No | 3485 (89.7) | 1130 (86.8) | 1156 (90.7) | 1199 (89.7) |  |
| Yes | 401 (10.3) | 172 (13.2) | 119 (9.3) | 110 (8.4) |  |
| Heart problems |  |  |  |  | 0.489 |
| No | 3122 (80.3) | 1060 (81.4) | 1017 (79.8) | 1045 (79.8) |  |
| Yes | 764 (19.7) | 242 (18.6) | 258 (20.2) | 264 (20.2) |  |
| Kidney problems |  |  |  |  | 0.024 |
| No | 3460 (89.0) | 1179 (90.6) | 1139 (89.3) | 1142 (87.2) |  |
| Yes | 426 (11.0) | 123 (9.4) | 136 (10.7) | 167 (12.8) |  |
| Liver problems |  |  |  |  | 0.480 |
| No | 3633 (93.5) | 1221 (93.8) | 1197 (93.9) | 1215 (92.8) |  |
| Yes | 253 (6.5) | 81 (6.2) | 78 (6.1) | 94 (7.2) |  |
| CESD-10 scale |  |  |  |  | <0.001 |
| <10 | 2542 (65.4) | 778 (59.8) | 846 (66.4) | 918 (70.1) |  |
| ≥10 | 1344 (34.6) | 524 (40.2) | 429 (33.6) | 391 (29.9) |  |

**SUPPLEMENTARY TABLE 2** The CESD-10 score comparison of the follow-up participants.

| **Items** | **Total (n=3886)** | **Q1 (n=1302)** | **Q2 (n=1275)** | **Q3 (n=1309)** | ***P* value** |
| --- | --- | --- | --- | --- | --- |
| Total score | 8.21±6.37 | 9.00±6.54 | 8.03±6.48 | 7.61±6.01 | <0.001 |
| Depresl | 0.85±1.07 | 1.00±1.10 | 0.80±1.06 | 0.74±1.02 | <0.001 |
| Effortl | 0.87±1.14 | 0.91±1.15 | 0.88±1.15 | 0.81±1.12 | 0.066 |
| Sleeprl | 1.04±1.20 | 1.14±1.20 | 1.06±1.23 | 0.92±1.16 | <0.001 |
| Whappyl | 1.09±1.20 | 1.17±1.20 | 1.00±1.17 | 1.10±1.22 | 0.001 |
| Flonel | 0.49±0.95 | 0.53±0.97 | 0.51±0.96 | 0.45±0.91 | 0.099 |
| Botherl | 0.87±1.10 | 1.04±1.16 | 0.83±1.09 | 0.74±1.03 | <0.001 |
| Goingl | 0.35±0.83 | 0.41±0.89 | 0.36±0.83 | 0.29±0.75 | 0.002 |
| Mindtsl | 0.88±1.11 | 0.97±1.13 | 0.83±1.11 | 0.82±1.09 | 0.001 |
| Fhopel | 1.45±1.29 | 1.42±1.28 | 1.43±1.29 | 1.49±1.29 | 0.409 |
| Fearl | 0.32±0.79 | 0.40±0.86 | 0.33±0.80 | 0.24±0.68 | <0.001 |

**SUPPLEMENTARY TABLE** **3** Logistic regression model on creatinine and depression of the follow-up participants.

| **Items** | **N** | **Events (%)** | **OR (95% CI)** | | | | |
| --- | --- | --- | --- | --- | --- | --- | --- |
|  |  |  | **Model 1** | **Model 2** | **Model 3** | **Model 4** | **Model 5** |
| Creatinine |  |  |  |  |  |  |  |
| High | 1309 | 391 (29.9) | Reference | Reference | Reference | Reference | Reference |
| Middle | 1275 | 429 (33.6) | 1.19 (1.01-1.41) | 1.08 (1.03-1.16) | 1.13 (0.95-1.34) | 1.22 (1.03-1.44) | 1.10 (1.02-1.26) |
| Low | 1302 | 524 (40.2) | 1.58 (1.35-1.86) | 1.19 (1.09-1.37) | 1.40 (1.17-1.67) | 1.64 (1.39-1.94) | 1.21 (1.04-1.35) |
| *P* for trend |  |  | <0.001 | 0.025 | 0.001 | <0.001 | 0.002 |

Model 1: Unadjusted model.

Model 2: Adjusted for demographic information.

Model 3: Adjusted for healthy related behaviors.

Model 4: Adjusted for disease histories.

Model 5: Fully adjusted model.

**
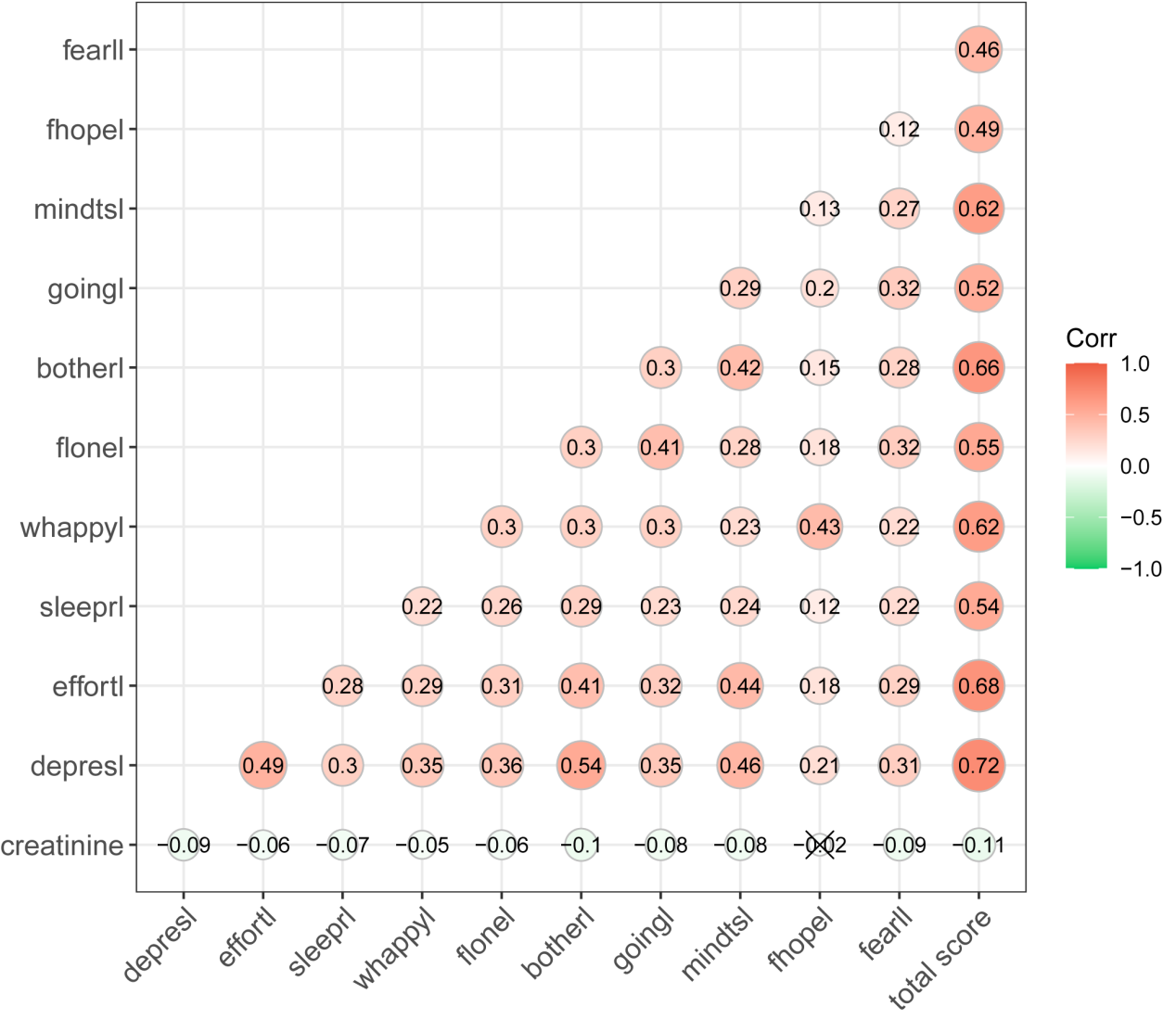
**

**SUPPLEMENTARY FIGURE 1** Correlation analysis between the creatinine and CESD-10 score.

**
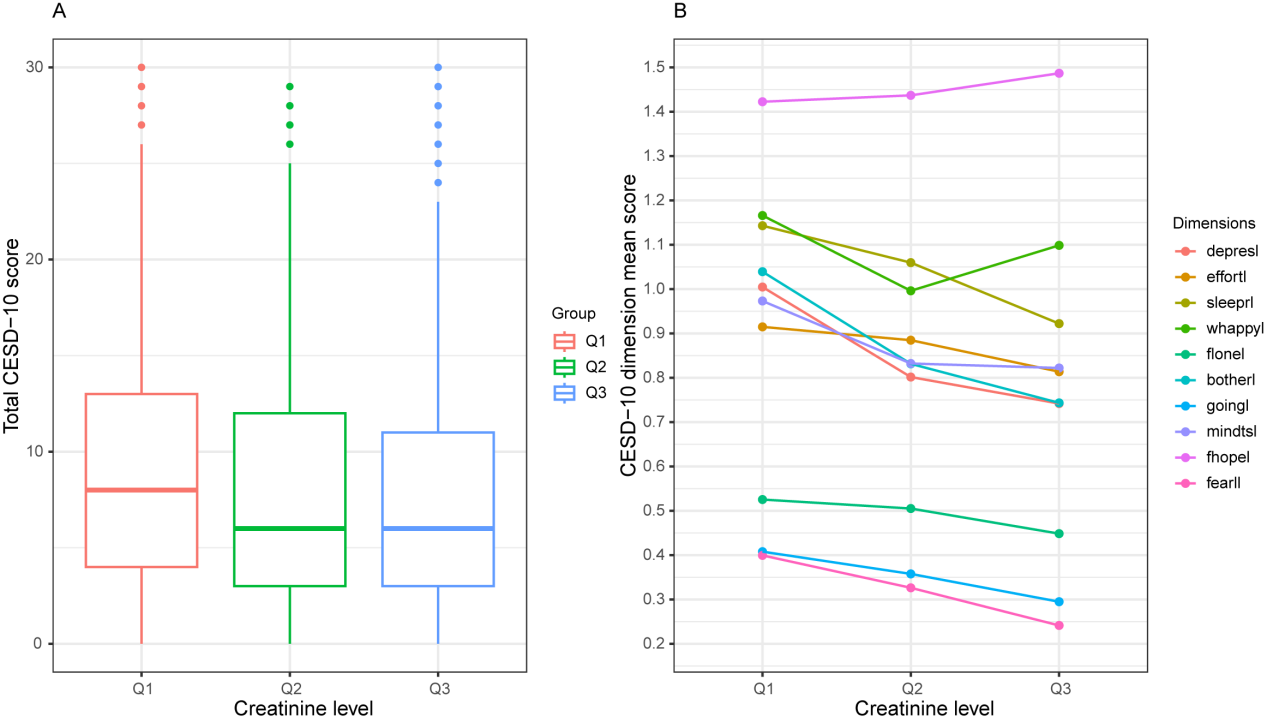
**

**SUPPLEMENTARY FIGURE 2** CESD-10 score between the groups of the follow-up participants. (A) The total CESD-10 score and creatinine level. (B) The dimension score of CESD-10 and the creatinine level. Abbreviations: CESD-10, 10-items center for epidemiological studies depression scale.

**
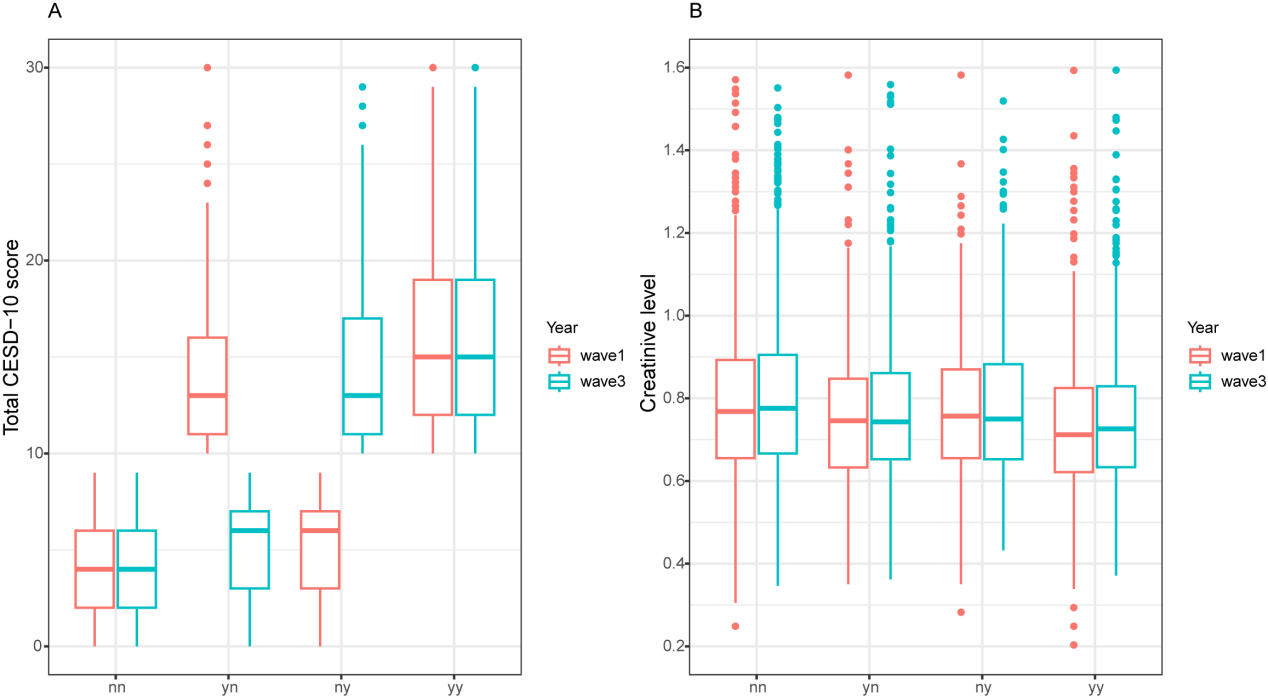
**

**SUPPLEMENTARY FIGURE 3** CESD-10 score and creatinine level comparison between the wave1 and wave3. (A) The total CESD-10 score between the four groups. (B) The creatinine level between the four groups. Abbreviations: nn, not depression both in wave1 and wave3; yn, depression in wave1, but not dep in wave3; ny, not depression in wave1, but depression in wave 3; yy, depression both in wave1 and wave3.


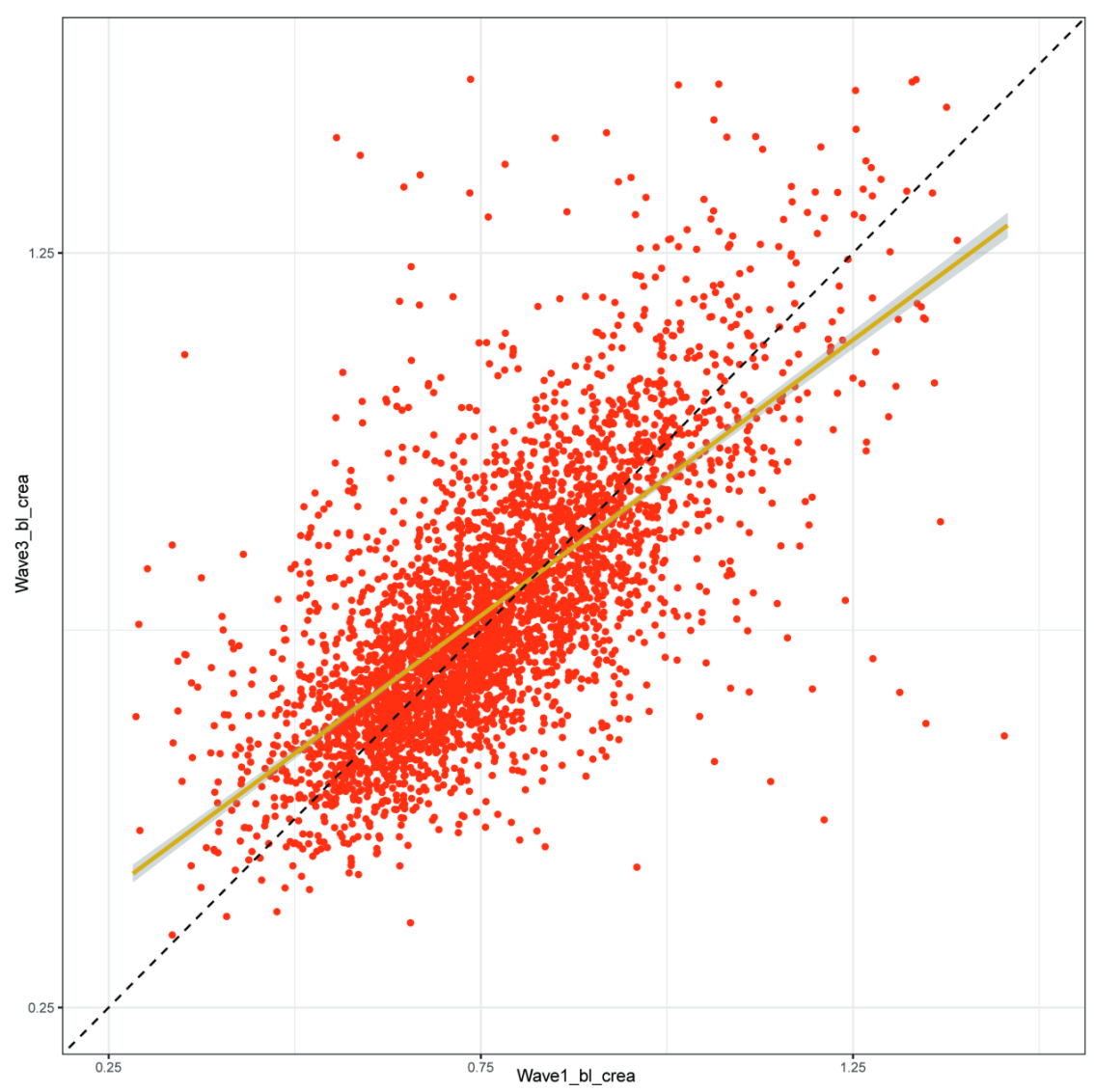


**SUPPLEMENTARY FIGURE 4** Correlation analysis between the creatinine concentration in Wave1 and Wave 3.


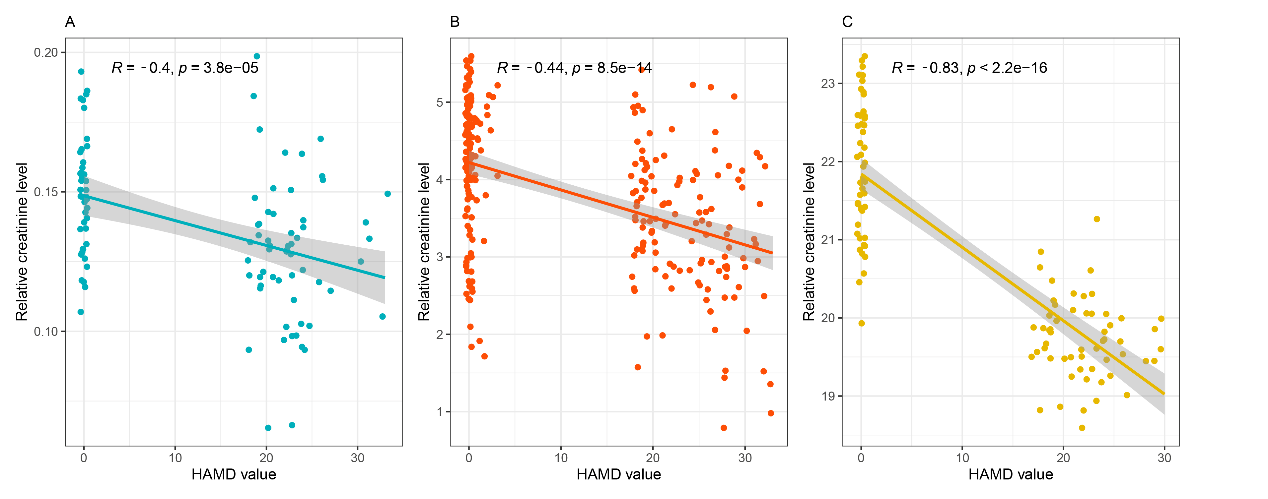


**SUPPLEMENTARY FIGURE 5** Correlation analysis between the creatinine and HAMD score of MDD patients from the MENDA. (A) The correlation between the plasma creatinine level and HAMD score in adults (PMID: 22239730). (B) The correlation between the urine creatinine level and HAMD score in adults (PMID: 23111923). (C) The correlation between the plasma creatinine level and HAMD score in children and adolescents (PMID: 29679072). Abbreviations: HAMD, hamilton depression scale.
